# Quality of AI-generated exercise awareness messages for older adults aligned with the ICFSR consensus: a comparative study of three LLMs

**DOI:** 10.1186/s11556-026-00428-8

**Published:** 2026-07-20

**Authors:** Yannick Laurent Tchenadoyo Bayala, Louis Sylvain Peng-wendé Ouedraogo, Abdoul Rahamane Cissé, Fulgence Kaboré, Aristide Relwendé Yameogo, Aboubakar Ouedraogo, Yamyellé Enselme Zongo, Wendlassida Joelle Stéphanie Zabsonré Tiendrébeogo, Dieu-Donné Ouedraogo

**Affiliations:** 1 Bogodogo University Hospital Center, Ouagadougou, Burkina Faso; 2 Tengandogo University Hospital Center, Ouagadougou, Burkina Faso

**Keywords:** Large language models, Health communication, Exercise prescription, Older adults, Readability assessment

## Abstract

**Background:**

Large Language Models (LLMs) are emerging as potential tools for health communication and patient education. However, their ability to translate complex medical guidelines into accessible, safe, and accurate messages for older adults remains insufficiently evaluated. To assess the capacity of ChatGPT-4, Claude AI, and Deepseek to generate exercise awareness messages for older adults, aligned with international expert consensus.

**Materials and methods:**

This was a cross-sectional observational study conducted between April and August 2025. Using the ICFSR 2021 Global Consensus on optimal exercise recommendations as the gold standard, we evaluated messages generated by three LLMs for 13 common chronic conditions in older adults. A standardized prompt was used to generate messages addressing exercise prescription considerations, disease progression, and recommended modalities. Two independent expert evaluators; a geriatrician and a sports medicine physician assessed five dimensions using a 5-point Likert scale; accuracy, clarity, safety, behavioral relevance, and absence of fabrication. Readability was measured using Flesch-Kincaid Grade Level (FKGL), Gunning Fog Index (GFI), and Simple Measure of Gobbledygook (SMOG). Inter-rater agreement was assessed using intraclass correlation coefficient (ICC).

**Results:**

Claude achieved the overall scores from both evaluators at 4.63 ± 0.55 and 4.60 ± 0.55, followed by ChatGPT-4 at 4.55 ± 0.50 and 4.48 ± 0.53 and Deepseek at 4.15 ± 0.87 and 4.17 ± 0.86. Inter-rater agreement was moderate was 0.668. Claude demonstrated accuracy scores at 4.58 ± 0.64, while ChatGPT-4 excelled in clarity with 5 ± 0. All models achieved perfect scores for absence of fabrication (5 ± 0). Readability indices revealed high complexity across all LLMs, with median FKGL values ranging from 10.84 to 10.93, corresponding to 10th-11th grade reading level, exceeding recommended levels for older populations. A significant correlation was found between Claude’s accuracy scores and FKGL (*r* = 0.599, *p* = 0.030).

**Conclusion:**

LLMs, particularly Claude, ChatGPT-4 and Deepseek demonstrate strong potential for generating accurate, safe, and hallucination-free exercise awareness messages for older adults. However, readability remains above recommended levels, requiring optimization.

**Supplementary Information:**

The online version contains supplementary material available at https://doi.org/10.1186/s11556-026-00428-8.

## Introduction

The aging of the global population is accompanied by a growing prevalence of chronic diseases and frailty syndromes [1]. Physical activity, and exercise in particular, represents a major means of prevention and management. The International Consensus on Optimal Exercise Recommendations for Promoting Healthy Longevity in Older Adults (ICFSR, 2021) established that exercise plays a central role in primary, secondary, and tertiary prevention by reducing the burden of comorbidities, functional dependence, and premature mortality [2, 3]. The effectiveness of exercise is explained by multifactorial effects on physiological capacity, psychological health, body composition, and the modulation of risk factors, making it an indispensable complement to pharmacological treatments [2].

At the same time, access to clear and tailored health information is essential to foster adherence of older adults to exercise prescriptions. However, the quality and readability of digital resources remain highly variable. In this context, the rise of artificial intelligence–based language models, particularly Large Language Models (LLMs), opens new perspectives [4]. Systems such as ChatGPT-4 (OpenAI), Claude AI (Anthropic), and Deepseek are now capable of producing health messages in natural language that are accessible to the general public [4, 5]. Nevertheless, the relevance, safety, and readability of the messages generated by these tools have not yet been rigorously evaluated when it comes to translating medical recommendations into messages adapted to older adults.

The research question of the present study is therefore as follows: to what extent can different language models generate exercise awareness messages tailored to the primary, secondary, and tertiary prevention of chronic diseases in older adults, in accordance with reference guidelines? We hypothesize that the quality and readability of the messages vary depending on the model used, and that some LLMs are more capable than others of faithfully translating guidelines into concrete advice.

The originality of this work lies in the systematic and comparative evaluation of several LLMs applied to the fields of geriatrics and sports medicine, with dual expert appraisal by a geriatrician and a sports physician, combined with standardized readability measures.

The objective of this study was to assess the ability of LLMs to generate awareness messages that are relevant, safe, and understandable for older adults, across different chronic conditions and levels of prevention, in order to explore their potential as complementary tools for translating guidelines into clinical practice.

## Materials and methods

### Design

This was a cross-sectional observational study designed to compare the ability of three large language models (LLMs) to generate physical activity awareness messages tailored to older adults. The study was conducted between April and August 2025. It was reported in accordance with the STROBE checklist and the QUEST framework (Quality of information, Understanding and reasoning, Expression style and persona, Safety and harm, Trust and confidence) for human evaluation of LLMs [6].

### Data source and recommendation selection

As the gold standard, we used the section *Role of exercise in primary*,* secondary*,* and tertiary disease prevention from the Global consensus on optimal exercise recommendations for enhancing healthy longevity in older adults* published by the International Conference on Frailty and Sarcopenia Research (ICFSR, 2021) (supplément 1), considered a reference standard in geriatric and sports medicine [2]. This consensus was chosen for its international scope. Thirteen common chronic conditions in older adults were selected: arthritis, cancer, chronic obstructive pulmonary disease, chronic kidney failure, heart failure, coronary artery disease, dementia, depression, osteoporosis, peripheral vascular disease, stroke, type 2 diabetes, and chronic venous insufficiency.

### LLMs selection and prompt design

Three LLMs were evaluated: ChatGPT-4o (OpenAI, San Francisco, USA), Claude 3.5 Sonnet (Anthropic, 2025), and Deepseek-R1 (Deepseek Inc., China). Queries were submitted via the web on July 15, 2025, in English. To avoid conversational memory bias, each condition was introduced in a new dialogue session. A standardized English prompt was used:

*“You are a health communication assistant specialized in geriatrics and exercise.*

*Please generate a short awareness message (max 150 words) for an older adult with Venous insufficiency, focusing on prevention.*

*Your message must:*

1. *Be written in clear, simple language that a person aged 65 + can easily understand.*
2. *Address explicitly three aspects:*
*Considerations for the exercise prescription.*
*How the disease may express itself and progress, and why exercise matters.*
*Recommended exercise modalities (types).*
3. *End with one motivating sentence encouraging the patient to take action safely.*
*Output: plain text only, ≤150 words, no bullet points, no references.”*

All generated responses were collected by a team member specialized in artificial intelligence (supplement 2).

### Performance evaluation

LLM performance was assessed by evaluating the quality of the generated messages for each condition and level of prevention. Five dimensions were considered, each reflecting a key aspect of health communication. The accuracy dimension reflects the degree of conformity of each generated message with the ICFSR recommendations summarized in the reference grid provided to evaluators, rather than a binary adherence score. This represents an expert judgment-based approximation of guideline adherence. Clarity assessed comprehensibility for older adults, taking into account potential limitations related to health literacy. Safety evaluated the absence of potentially harmful instructions or recommendations inconsistent with best medical practice. Behavioral relevance was defined as the ability of the message to encourage older adults to adopt or maintain appropriate physical activity. Finally, absence of fabrication identified any invented or erroneous information that could mislead patients.

Each dimension was scored on a five-point Likert scale, ranging from 1 (minimal quality) to 5 (maximal quality) [7, 8]. Two physicians; a geriatrician and a sports medicine physician; independently rated all responses, each with at least three years of clinical experience in their respective fields. The raters were blinded to the identity of the LLM that had generated the responses to minimize assessment bias.

### Readability

The readability of the generated messages was assessed using three widely applied indices in the evaluation of patient-facing medical texts: Flesch-Kincaid Grade Level (FKGL), Gunning Fog Index (GFI), and Simple Measure of Gobbledygook (SMOG) [9–11]. FKGL estimates the minimum grade level required to understand a text at first reading [9]. The GFI focuses on sentence length and the proportion of complex words, indicating the years of education needed for comprehension [10]. The SMOG, designed specifically for health communication, estimates grade level based on the density of polysyllabic words [11]. All indices were calculated using standard formulas through an online readability calculator.

$$FKGL=0.39*\frac{words}{sentences}+11.8*\frac{syllabes}{words}-15.59$$

$$GFI=0.4*\left(\frac{words}{sentences}+100*\frac{complex\:words}{words}\right) $$

$$SMOG=1.043*\sqrt{30*\frac{polysyllabic\:words}{sentences}}+3.1291$$

### Statistical analysis

Scores were expressed as mean ± standard deviation and median (interquartile range). Inter-model comparisons were performed using repeated-measures ANOVA or the Friedman test with Wilcoxon post-hoc comparisons for non-parametric data. Inter-rater agreement was measured using the intraclass correlation coefficient (ICC) with a mixed-effects model. Thresholds for interpretation were: excellent (ICC ≥ 0.90), good (0.75–0.90), moderate (0.50–0.75), and poor (< 0.50) [12]. Inter-model comparisons were performed using the Kruskal-Wallis test, followed by post-hoc pairwise Wilcoxon signed-rank tests with Bonferroni correction. Effect sizes were calculated as r = Z/√N, where values of 0.1, 0.3, and 0.5 indicate small, moderate, and large effects, respectively. Analyses were conducted in R (version 4.5.1), with statistical significance set at *p* < 0.05.

### Ethical considerations

The study did not involve any real human participants and was limited to the analysis of AI-generated text. In accordance with local regulations, the institutional ethics committee exempted this work from the requirement for ethical approval.

## Results

### Comparison of AI models

The overall scores assigned by the geriatrician were 4.55 ± 0.50 for ChatGPT-4, 4.63 ± 0.55 for Claude, and 4.15 ± 0.87 for Deepseek. For the sports medicine physician, they were 4.48 ± 0.53, 4.60 ± 0.55, and 4.17 ± 0.86, respectively. The ICC between the two evaluators was 0.668, IC 95% [-0.074 ; 0.790], *p* = 0.044, indicating moderate agreement. Table 1 presents the performance scores of AI models according to geriatrician and sports medicine physician evaluations.

**Table 1:** Performance scores of AI models according to geriatrician and sports medicine physician evaluations (Mean ± SD)

AI Model	Accuracy	Clarity	Safety	Relevance	Hallucination-Free	Overall Score
Geriatrician						
ChatGPT-4	4.15 ± 0.38	5 ± 0	4.46 ± 0.52	4.15 ± 0.38	5 ± 0	4.55 ± 0.50
Claude	4.62 ± 0.65	4 ± 0	4.85 ± 0.55	4.69 ± 0.48	5 ± 0	4.63 ± 0.55
Deepseek	3.31 ± 0.63	4.92 ± 0.28	4.23 ± 0.44	3.31 ± 0.63	5 ± 0	4.15 ± 0.87
Sports medicine physician						
ChatGPT-4	4 ± 0.41	5 ± 0	4.23 ± 0.44	4.15 ± 0.38	5 ± 0	4.48 ± 0.53
Claude	4.54 ± 0.66	4 ± 0	4.77 ± 0.60	4.69 ± 0.48	5 ± 0	4.60 ± 0.55
Deepseek	3.31 ± 0.63	5 ± 0	4.15 ± 0.38	3.38 ± 0.65	5 ± 0	4.17 ± 0.86

Friedman test ; χ²(2) = 12.154, *p* = 0.002

**Fig. 1:**
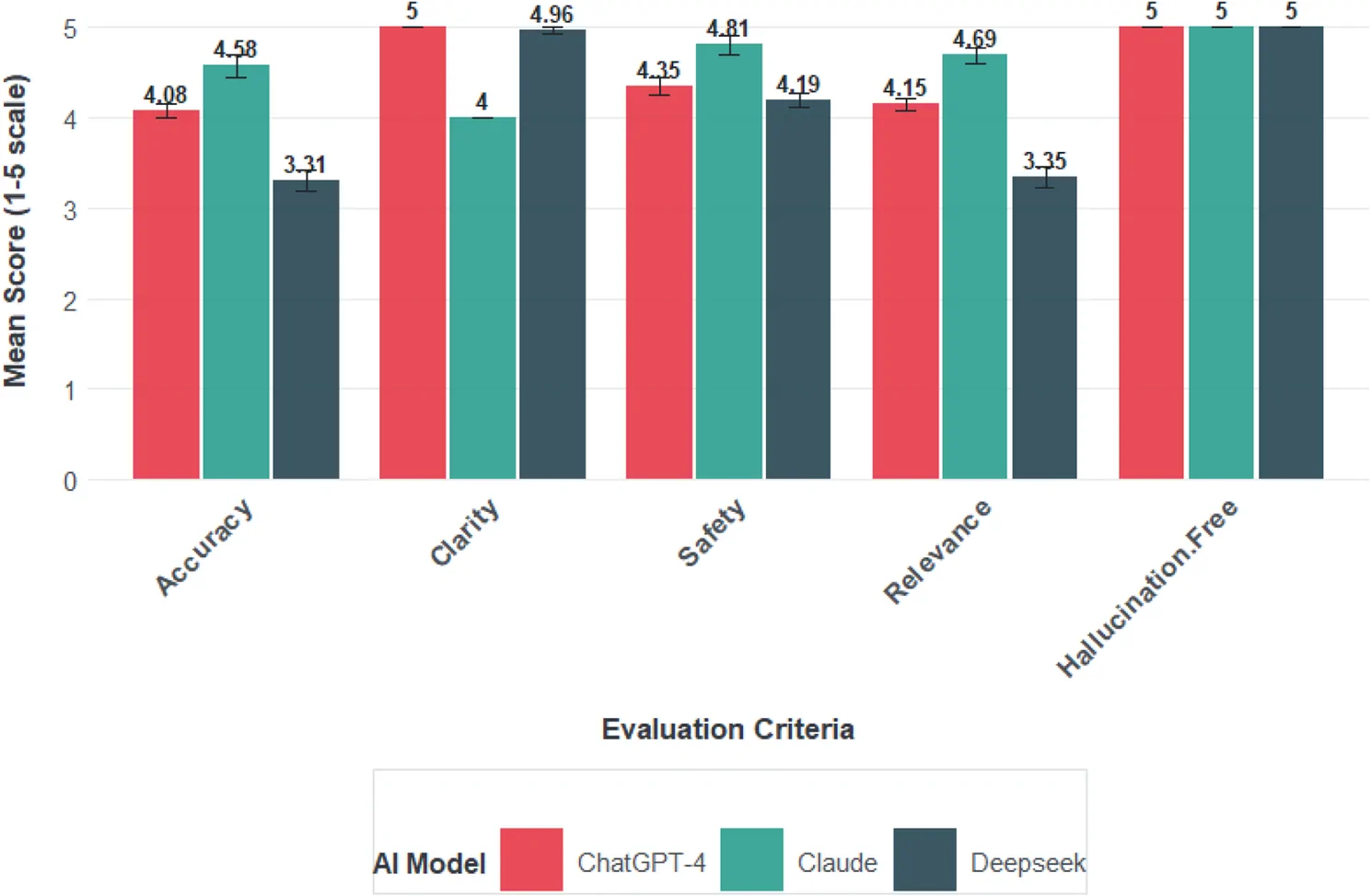
Grouped bar chart illustrating the five dimensions evaluated across LLMs

Combined scores showed a mean accuracy of 4.08 ± 0.39 for ChatGPT-4, 4.58 ± 0.64 for Claude, and 3.31 ± 0.62 for Deepseek. All models achieved a maximum score for hallucination-free responses, with 5 ± 0 in all evaluations. The overall mean scores across all dimensions for each LLM are illustrated in Fig. 1.

Overall scores differed significantly across the three models (Kruskal-Wallis: χ² = 14.33, df = 2, *p* < 0.001). Post-hoc Dunn’s test with Bonferroni correction revealed that ChatGPT-4 scored significantly higher than Deepseek (*p* = 0.021, *r* = 0.773) and Claude scored significantly higher than Deepseek (*p* < 0.001, *r* = 0.627). No significant difference was observed between ChatGPT-4 and Claude (*p* = 0.311, *r* = 0.419).

### Comparative analysis by pathology

**Fig. 2:**
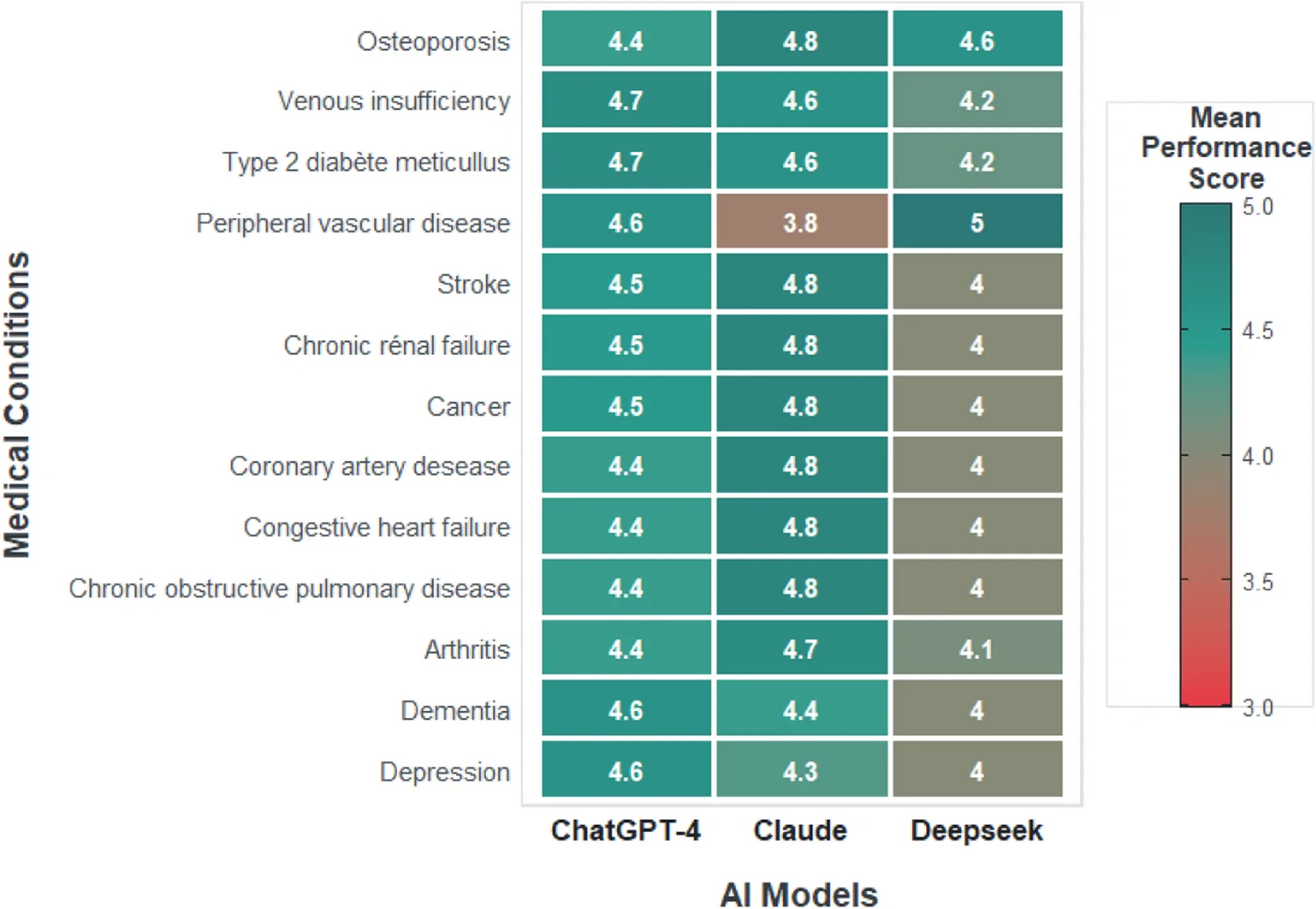
Heatmap of mean values by pathology for each LLM

The mean scores by pathology were 4.6 ± 0.48 for osteoporosis and 4.5 ± 0.56 for venous insufficiency and type 2 diabetes mellitus. Figure 2 presents the heatmap of mean scores obtained for each LLM by pathology.

### Readability

**Fig. 3:**
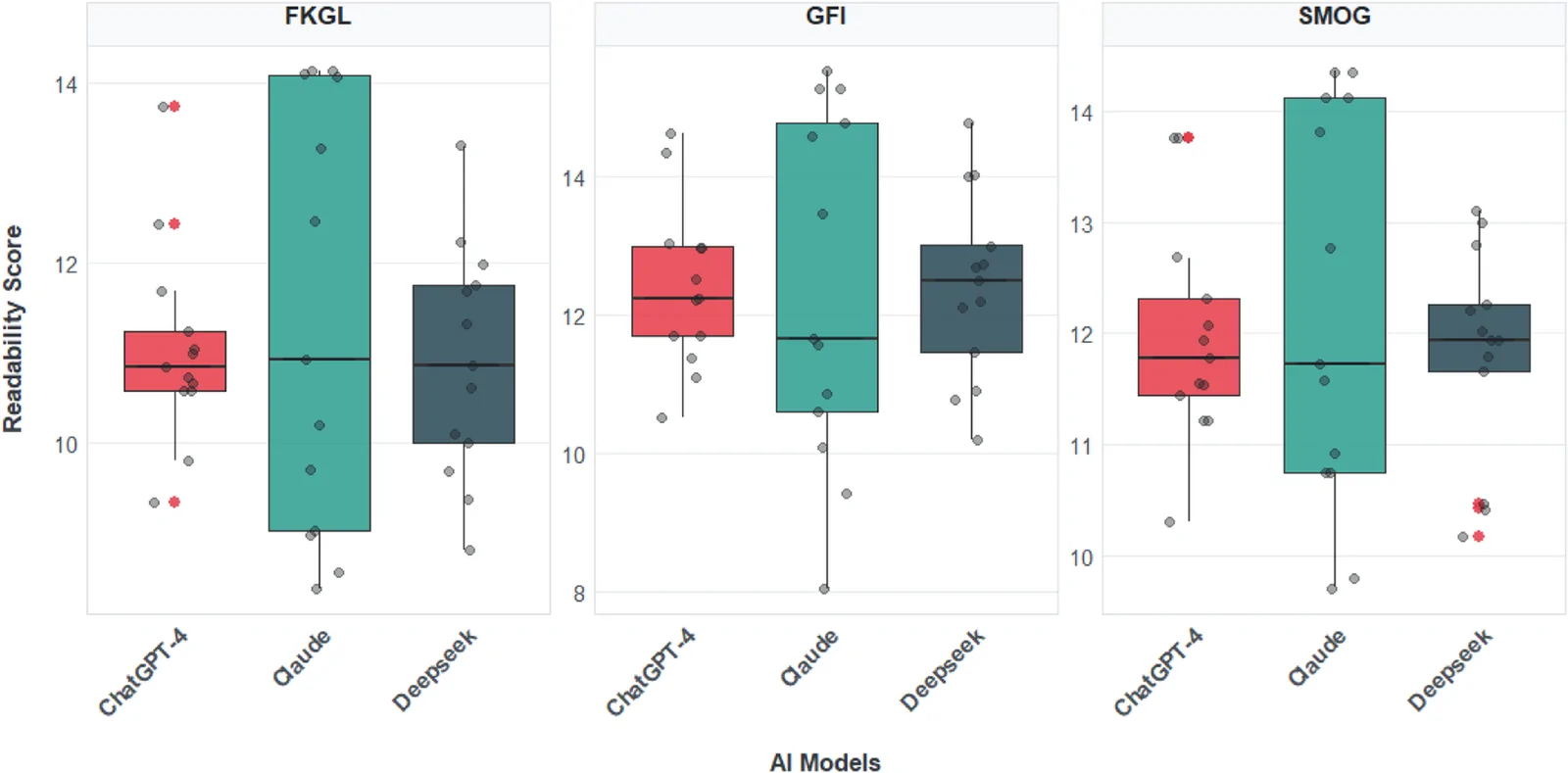
Boxplot of readability scores across LLMs

For the FKGL index, median values were 10.84 [10.59–11.25] for ChatGPT-4, 10.93 [9.03–14.07] for Claude, and 10.86 [10.01–11.76] for Deepseek. Regarding the GFI index, medians were 12.23 [11.7–12.98] for ChatGPT-4, 11.66 [10.61–14.77] for Claude, and 12.49 [11.46–13] for Deepseek. For the SMOG index, median values were 11.78 [11.44–12.31] for ChatGPT-4, 11.73 [10.75–14.12] for Claude, and 11.94 [11.66–12.26] for Deepseek. Figure 3 depicts the boxplot of positional and dispersion parameters by LLM and index.

**Fig. 4:**
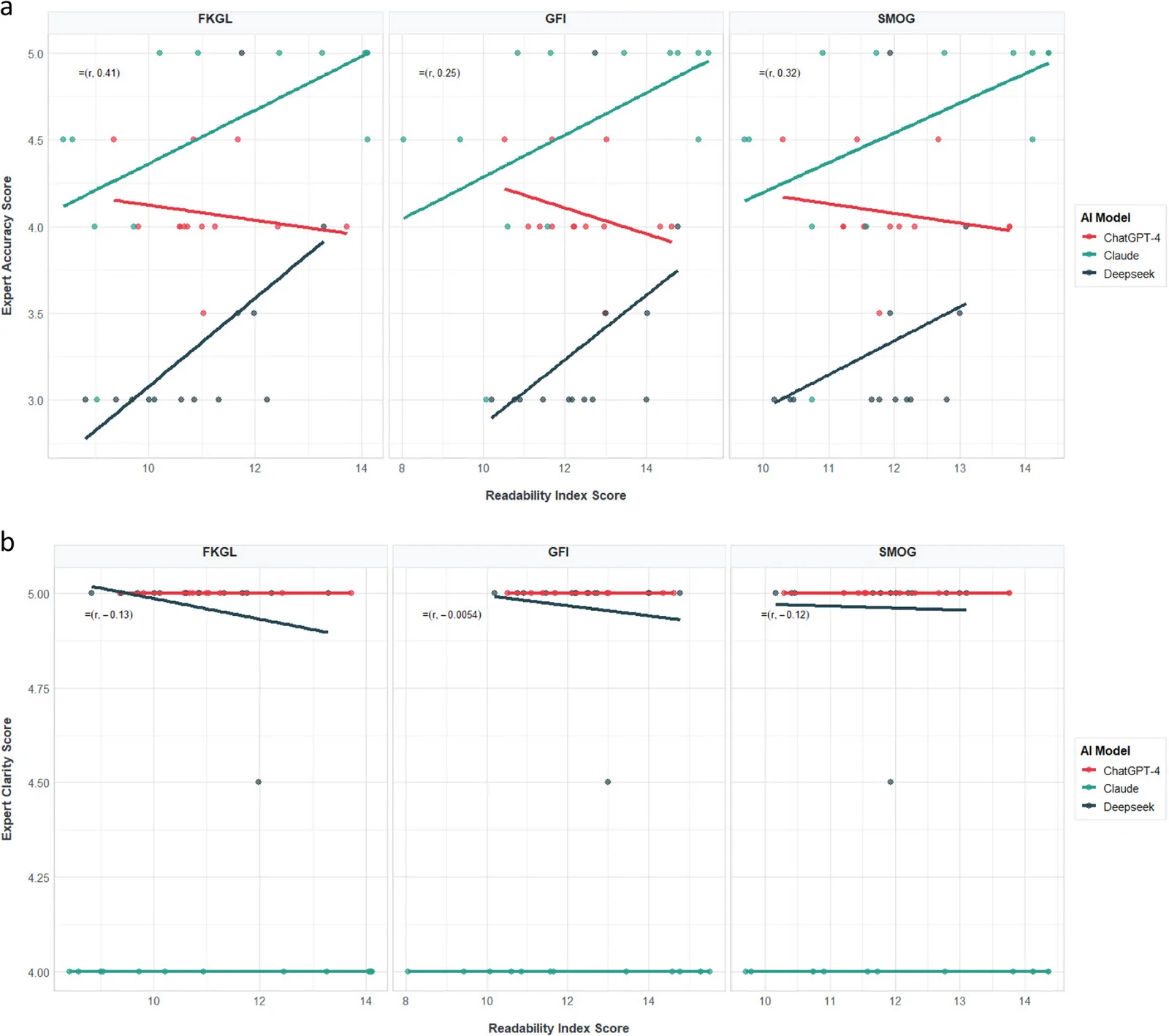
**a** Pearson correlations between expert accuracy ratings and automated readability indices. Pearson correlations between expert accuracy ratings and automated readability indices. FKGL: ChatGPT-4 (*p* = 0.571), Claude (*p* = 0.030), Deepseek (*p* = 0.053); GFI: ChatGPT-4 (*p* = 0.283), Claude (*p* = 0.075), Deepseek (*p* = 0.149); SMOG: ChatGPT-4 (*p* = 0.517), Claude (*p* = 0.080), Deepseek (*p* = 0.297). **b** Pearson correlations between expert clarity ratings and automated readability indices. Pearson correlations between expert clarity ratings and automated readability indices. Correlations were not computable for ChatGPT-4 and Claude due to zero variance in clarity scores. Deepseek — FKGL (*p* = 0.406), GFI (*p* = 0.671), SMOG (*p* = 0.297)

Pearson correlation reached statistical significance, including Claude-FKGL (*r* = 0.599, *p* = 0.030). Figure 4 shows the correlations between expert accuracy and clarity ratings and automated readability indices for the three AI models.

## Discussion

### Main findings

This study evaluated the ability of three LLMs to generate exercise awareness messages for older adults. Claude achieved the highest overall scores (4.63 ± 0.55 and 4.60 ± 0.55), followed by ChatGPT-4 (4.55 ± 0.50 and 4.48 ± 0.53) and Deepseek (4.15 ± 0.87 and 4.17 ± 0.86). The complete absence of hallucinations across all models represents a promising outcome for their use in healthcare. Readability scores were comparable across the three LLMs, with median FKGL values ranging from 10.8 to 10.9, corresponding to a high school reading level.

Our findings are consistent with recent literature showing the superiority of Claude in terms of completeness and relevance in medical scenarios [13]. A comparative study reported that Claude achieved higher DISCERN scores (47.6 ± 1.2) than other chatbots for kidney stone–related information, supporting our observations regarding accuracy [14]. The FKGL readability levels observed here are similar to those reported in other studies evaluating LLMs in healthcare [15]. However, the generated messages still exceed the recommended levels for older populations.

### Comparative performance of LLMs

LLMs have demonstrated their potential to enhance patient education and empowerment by personalizing medical information [16]. In our study, Claude had the highest accuracy, with an overall mean score of 4.58 ± 0.64 compared with ChatGPT-4 (4.08 ± 0.39) and Deepseek (3.31 ± 0.62). This difference suggests that Claude more faithfully translates ICFSR recommendations into practical advice. This may be explained by its use of Reinforcement Learning from Human Feedback (RLHF), which strengthens factual fidelity and reduces hallucinations [17]. Another contributing factor is Claude’s larger context window, which facilitates the faithful integration of ICFSR guideline recommendations, thereby improving the precision of generated advice [18].

By contrast, ChatGPT-4 obtained the highest clarity score (5 ± 0), likely due to its architecture favoring simplified writing style, short sentences, and accessible vocabulary, as reported in other medical studies [6]. This tendency to standardize language and reduce linguistic variability contributes to greater comprehensibility for older adults. Safety was consistently high across all LLMs, reflecting their design with integrated safeguards. For instance, Claude incorporates Anthropic’s Constitutional AI framework, specifically developed to reduce hallucinations and improve factual reliability, consistent with recent analyses of LLM trustworthiness [19].

The relative weakness of Deepseek in accuracy may be attributed to less stringent RLHF compared with OpenAI and Anthropic, limiting its ability to align medical outputs strictly with evidence-based guidance [17, 18]. Moreover, although Deepseek integrates powerful generative models, recent comparative analyses indicate greater variability and tendencies toward oversimplification, potentially compromising factual accuracy when translating clinical recommendations.

### Readability and accessibility

All three LLMs produced messages at a 10th–11th grade reading level (FKGL 10.84–10.93), substantially exceeding the 6th–8th grade threshold recommended for patient-facing health materials, which represents a meaningful limitation for clinical deployment given the high prevalence of low health literacy among older adults [9]. It should be noted, however, that lexical readability indices capture surface-level textual complexity but do not directly measure comprehension, cognitive load, or motivational impact in the target population. Prompt optimization, through explicit constraints on sentence length and vocabulary, represents a practical and immediate avenue for improvement, and any clinical use of AI-generated messages should systematically include a readability verification step prior to dissemination.

The GFI, based on sentence length and proportion of complex words, showed even higher values (11.66–12.49), suggesting that 12 years of formal education are required for first-pass comprehension. The SMOG index, specifically designed for health communication and based on polysyllabic word density, yielded medians between 11.73 and 11.94, further confirming the lexical complexity of the generated messages.

These values greatly exceed the recommended grade 6–8 reading level for health information targeting the general population [10]. Low health literacy is particularly common among older adults, making content adaptation essential [20].

The significant correlation between Claude’s accuracy scores and FKGL (*r* = 0.599, *p* = 0.030) may be explained by the fact that slightly higher readability scores favor terminological precision and fidelity to guideline content, particularly in specialized medical texts where over simplification may reduce accuracy. This relationship is consistent with prior studies showing that moderate linguistic complexity, as measured by FKGL, is associated with better transmission of clinical nuances and reduced information loss in AI-generated messages [21].

The convergence of all three indices at elevated levels suggests a systematic issue rather than a methodological artifact. The similarity across models indicates that linguistic complexity is inherent to the translation of specialized medical content into patient-facing messages [16]. To improve readability, prompts could be refined to explicitly require lower-grade levels, shorter sentences, and avoidance of polysyllabic terms. Incorporating concrete examples rather than abstract concepts could also enhance comprehension.

### Practical implications

LLMs may serve as complementary tools for generating personalized educational materials in geriatrics. By analyzing and producing large amounts of textual data, they hold significant potential for patient education [6]. They may have the potential to assist clinicians in translating guidelines into patient-oriented advice, though this hypothesis requires prospective evaluation before any clinical deployment.

However, these tools should not replace clinical expertise. Expert human oversight remains essential in the application of LLMs to healthcare [6]. Physicians must review, adapt, and contextualize each message according to the patient’s characteristics. The absence of hallucinations in our study does not guarantee absolute reliability across all clinical contexts.

For clinical implementation, user interfaces enabling healthcare professionals to rapidly generate, review, and customize messages are needed. Training for clinicians in the critical use of these tools would also be essential.

### Study limitations

This study has several limitations. First, the evaluation covered only 13 pathologies, which limits generalizability. Second, the moderate inter-rater agreement (ICC = 0.668) indicates variability in expert judgments, reflecting the subjective nature of certain assessed dimensions [12]. Third, a critical limitation of this study is the complete absence of validation with older adults. The evaluation relied exclusively on expert judgment and automated readability indices, which are surrogate measures of comprehension and do not capture the perspective of the intended recipients. Health literacy varies considerably among older adults and is not fully captured by lexical readability scores alone; a message may achieve satisfactory expert ratings while remaining poorly understood or insufficiently motivating for patients with low health literacy. As such, the clinical and behavioral relevance of the generated messages cannot be confirmed without end-user studies incorporating cognitive debriefing, comprehension testing, and motivational assessments with the target population.

Fourth, all messages were generated in English, restricting applicability to other linguistic and cultural contexts. LLM performance is constrained by the quality of input data, and prompt formulation strongly influences results. Our choice of a standardized prompt, while methodologically rigorous, may not represent the optimal use of each model.

Five, lexical readability indices such as FKGL, GFI, and SMOG measure surface-level textual complexity based on word and sentence length, but do not capture cognitive load, logical structure, or motivational tone dimensions that are directly relevant to health communication effectiveness in older adults. Furthermore, motivational tone, a critical component of behavioral health communication, was assessed only indirectly through expert ratings of “behavioral relevance,” which itself constitutes a proxy measure subject to subjectivity.

Finally, LLMs evolve rapidly, and our findings reflect the versions tested in July 2025. Later releases may display different performance.

### Research perspectives

Several avenues merit further exploration. Validation with older patients is needed to assess comprehension, acceptability, and motivational impact. Eye-tracking studies or cognitive load analyses could clarify linguistic difficulties. A multilingual extension could determine whether performance varies across languages, while exploring other LLMs and updated versions would broaden understanding of the evolving technological ecosystem.

Future studies should complement lexical readability indices with validated comprehensive tools such as the Patient Education Materials Assessment Tool (PEMAT) or plain language checklists, which additionally assess comprehensibility, actionability, and motivational tone. Cognitive load analyses or eye-tracking studies could further elucidate the specific linguistic barriers faced by older adults when processing AI-generated health messages.

Prompt optimization through iterative prompting or few-shot learning could improve both accuracy and readability. Finally, randomized controlled trials evaluating the impact of AI-generated messages on treatment adherence, physical activity, and health outcomes in older adults will be necessary to establish their clinical utility.

## Conclusion

This study demonstrates that LLMs; particularly Claude, ChatGPT-4, and Deepseek can generate relevant exercise awareness messages for older adults with good accuracy, clarity, safety, and without hallucinations. However, readability remains above recommended thresholds, requiring improvement. These tools represent a promising complement for clinicians in translating guidelines into practical advice, provided that rigorous medical oversight is maintained and further research addresses their optimization and validation in target populations. These findings should be interpreted as preliminary evidence supporting further development, and end-user validation with older adults remains an indispensable step before any clinical deployment of AI-generated exercise messages.

## Supplementary Information

Supplementary Material 1.

Supplementary Material 2.

## Data Availability

No datasets were generated or analysed during the current study.
