# Supplementary material for: Quality of AI-generated exercise awareness messages for older adults aligned with the ICFSR consensus: a comparative study of three LLMs
Source: Eur Rev Aging Phys Act. 2026 Jul 20;23:38. doi: 10.1186/s11556-026-00428-8 (PMC13673795; doi:10.1186/s11556-026-00428-8)
Supplement: Supplementary file 1 [file 11556_2026_428_MOESM2_ESM.pdf]

Table 5
Role of Exercise in Primary, Secondary and Tertiary Disease Prevention.

Disease	Postulated mechanisms of exercise effect on disease prevention	Considerations for the prescription for secondary and tertiary prevention (disease expression and progression)	Recommended exercise modality
Arthritis (osteoarthritis for prevention; all arthritis for treatment)	• Decreased body weight<br>• Maintenance of cartilage integrity<br>• Maintenance of muscle and tendon strength	• Low impact<br>• Sufficient volume to achieve a healthy weight if obese<br>• Sufficient intensity to maintain muscle and tendon strength	• Aerobic exercise<br>• Resistance exercise
Cancer (breast, colon, prostate)	• Decreased body fat<br>• Decreased oestrogen levels<br>• Decrease in gastrointestinal transit time<br>• Increased prostaglandin F2<br>• Improved immune function and reduced inflammation	• Resistance training with dietary intervention may offset myopathy and reduce prevalence of cancer cachexia	• Aerobic exercise<br>• Resistance exercise
Chronic obstructive pulmonary disease	• Increased adherence to smoking cessation, dietary behaviours<br>• Increased muscle mass<br>• Improved lung function	• Resistance training may be more tolerable in severe disease, and addresses cachexia or COPD<br>• Combined effects complementary if feasible<br>• Time exercise sessions to coincide with bronchodilator medication peak.<br>• Use oxygen during aerobic exercise as needed	• Aerobic exercise<br>• Resistance exercise
Chronic renal failure	• Reduced risk of hypertension<br>• Reduced risk of type 2 diabetes mellitus	• Exercise reduces cardiovascular and metabolic risk factors, improves depression<br>• Resistance training offsets myopathy of chronic renal failure	• Aerobic exercise<br>• Resistance exercise
Congestive heart failure	• Decreased risk of ischaemic heart disease<br>• Decreased risk of hypertension<br>• Decreased risk of type 2 diabetes mellitus<br>• Decreased risk of visceral obesity	• Improves cardiovascular function and contractility, morphology<br>• Improves hypertension and lipid profile, inflammation, visceral obesity, risk of secondary ischemic events<br>• Resistance training addresses cardiac cachexia<br>• Exercise improves psychological health	• Aerobic exercise<br>• Resistance exercise[a]
Coronary artery disease	• Decreased blood pressure<br>• Decreased LDL cholesterol<br>• Increased HDL cholesterol<br>• Decreased fibrinogen<br>• Decreased total body fat, visceral fat<br>• Decreased insulin resistance, hyperinsulinemia<br>• Decreased cortisol levels, inflammatory cytokines<br>• Increased adherence to smoking cessation, dietary behaviours<br>• Decreased depression, anxiety<br>• Improved endothelial cell function<br>• Increased aerobic capacity	• Exercise improves all risk factors to prevent recurrent events<br>• Exercise improves aerobic capacity which is linked to lower mortality<br>• Complementary effects on exercise capacity and metabolic profile from combined exercise modalities<br>• Resistance training may be more tolerable if the ischaemic threshold is very low due to lower heart rate response to training	• Aerobic exercise<br>• Resistance exercise
Dementia	• Improved cerebral blood flow<br>• Increased neurotrophic factors in CNS<br>• Hippocampal neurogenesis<br>• Anabolic hormones<br>• Prevention of diabetes/insulin resistance<br>• Prevention of stroke<br>• Prevention of hypertension<br>• Prevention and treatment of depression, anxiety and insomnia	• Exercise under supervision if cognition is moderately to severely impaired<br>• Attenuation of cardiometabolic risk factors for progression<br>• Improved functional independence<br>• Reduced falls risk with resistance and balance exercise	• Aerobic<br>• exercise<br>• Balance/gait/functional exercise<br>• Resistance exercise

(continued on next page)

Table 5 ***(continued)***

Disease	Postulated mechanisms of exercise effect on disease prevention	Considerations for the prescription for secondary and tertiary prevention (disease expression and progression)	Recommended exercise modality
	• Avoidance of head trauma during exercise is critical		
Depression	• Increased self-efficacy, mastery<br>• Internalised locus of control<br>• Decreased anxiety<br>• Improved sleep<br>• Increased self-esteem<br>• Increased social engagement, decreased isolation<br>• Decreased need for drugs associated with depression (beta blockers, alpha blockers, sedative hypnotics)<br>• Decreased body fat, improved body image	• High-intensity resistance training and adequate volumes of aerobic exercise are more efficacious than low-intensity/low-volume exercise in major depression	• Aerobic exercise<br>• Resistance exercise<br>• Yoga/other mind-body exercise
Osteoporosis / Osteoporotic fracture	• Increased bone density<br>• Increased tensile strength<br>• Increased muscle mass<br>• Improved gait stability and balance<br>• Improved nutritional intake (energy, protein, calcium, vitamin D)<br>• Reduced fear of falling, improved self-efficacy<br>• Increased overall activity levels, mobility<br>• Decreased need for drugs associated with postural hypotension, falls, hip fractures (antidepressants, antihypertensives, sedative-hypnotics)	• High-impact, high-velocity activity (e.g., jumping) is potent if tolerable; avoid if osteoarthritis is present.<br>• Resistance training effects are local to muscles contracted.<br>• Balance training should be added to prevent falls and must be challenging	• High-impact exercise<br>• Progressive resistance training<br>• Balance training
Peripheral vascular disease	• Prevention of hypertension<br>• Prevention of diabetes<br>• Improved lipid profile<br>• Assistance in smoking cessation<br>• Reduction in adiposity/visceral adiposity	• Vascular effect is systemic; upper limb ergometry may be substituted for leg exercise if necessary<br>• Resistance training has a similar effect on claudication as aerobic exercise<br>• Low-intensity resistance training is ineffective.<br>• Exercise only to the onset /early phase of claudication; rest and repeat	• Aerobic exercise<br>• Resistance exercise
Stroke	• Decreased obesity<br>• Decreased cholesterol<br>• Prevention of diabetes<br>• Prevention of hypertension<br>• Prevention of atrial fibrillation	• Start with resistance and balance training until ambulation is safe<br>• Cognitive impairment may require close supervision<br>• Avoid Valsalva and breath holding to minimise hemodynamic excursions	• Aerobic exercise<br>• Resistance exercise<br>• Balance and functional training
Type 2 diabetes mellitus	• Improved insulin sensitivity<br>• Increased GLUT-4 protein and translocation to membrane sites<br>• Reduced visceral fat mass<br>• Decreased cortisol response to stress<br>• Improved lipid profile<br>• Decreased blood pressure<br>• Increased muscle mass	• Exercise at least every 48 h to optimise glucose regulation<br>• May need to avoid impact exercises if peripheral neuropathy present<br>• Monitor blood glucose before and after exercise if not well-controlled	• Aerobic exercise<br>• Resistance exercise
Venous insufficiency	• Increased muscle mass/strength lower limbs<br>• Decreased adiposity	• Local muscle contractions in calf stimulate the return of fluid via the lymphatic system<br>• Utilise lower body training; elevate legs when possible	• Aerobic exercise<br>• Resistance exercise

[a] Indicates that the modality of exercise has been shown to affect the postulated mechanistic factors but has not yet been shown to prevent distal disease outcomes in epidemiological/clinical studies.
